# Oropharyngeal colostrum administration in neonates with gastroschisis: a randomized clinical trial

**DOI:** 10.5935/2965-2774.20230010-en

**Published:** 2023

**Authors:** Hellen Porto Pimenta, Adriana Duarte Rocha, Aline Carnevale Lia Dias Guimarães, Ana Carolina Carioca da Costa, Maria Elisabeth Lopes Moreira

**Affiliations:** 1 Instituto Fernandes Fiqueira - Rio de Janeiro (RJ), Brazil

**Keywords:** Human milk, Colostrum, Immune therapy, Gastroschisis

## Abstract

**Objective:**

To evaluate the effect of colostrum therapy on days to start a suckling diet in newborns diagnosed with simple gastroschisis.

**Methods:**

Randomized clinical trial with newborns diagnosed with simple gastroschisis at a federal hospital in Rio de Janeiro who were randomized to receive oropharyngeal administration of 0.2mL of colostrum or a “sham procedure” during the first 3 days of life. The analysis included clinical outcomes such as days without food, days with parenteral feeding, days until the start of enteral feeding, days to reach complete enteral feeding, sepsis and length of hospital stay.

**Results:**

The onset of oral feeding (suction) in patients with simple gastroschisis in both groups occurred at a median of 15 days.

**Conclusion:**

The present study showed that there were no significant differences in the use of colostrum therapy and the number of days to the start of enteral feeding and suction diet between groups of newborns with simple gastroschisis.

## INTRODUCTION

The oropharyngeal administration of colostrum, popularly known as colostrum therapy, uses raw colostrum dispensed directly into the oral mucosa of very low birth weight preterm infants in small volumes. It interacts with lymph tissue and permeates the oral cavity into the oropharynx, activating the immune system and modulating the inflammatory response of newborns (NBs).^(1-4)^

It is an outstanding therapeutic resource because it provides better neonatal outcomes in NBs who are subject to complications inherent to preterm birth or acquired in the hospital environment.^(3,4)^

Colostrum is the secretion produced by the mammary gland at the end of pregnancy and in the first days after delivery and contains numerous immunological components transported from the maternal circulation. Some studies suggest that the earlier the delivery, the longer it takes to close the junctions between the alveolar cells of the mammary gland, allowing greater passage of immune components into the colostrum, which may have biological significance for the protection of the NB against infections.^(5-7)^

Studies proposing the oropharyngeal administration of colostrum in neonatal units involving preterm NBs have not included those with gastroschisis.^(8-14)^

Newborns with gastroschisis remain several days without feeding or oral experiences and are dependent on parenteral feeding. In most cases, the initiation of feeding is delayed due to reactions to food (vomiting, abdominal distension, stasis, increased gastric residues, among others). Clinical conditions associated with infection in the catheter used for parenteral feeding and other neonatal conditions also contribute to feeding delay.^(15,16)^

Thus, this study aimed to evaluate the effect of colostrum therapy on the number of days from birth to the beginning of a suckling diet in NBs diagnosed with simple gastroschisis. The secondary clinical outcomes were to evaluate the effects of this intervention on nutritional aspects (days on zero feeding, days on parenteral nutrition, days to start enteral diet and days to achieve full feeding), sepsis and length of hospital stay.

## METHODS

A double-blind randomized trial was conducted with newborns at a federal public children’s hospital in Rio de Janeiro that specializes in preterm NBs and children with surgical problems. Newborns diagnosed with simple gastroschisis who were born at the hospital and admitted to the neonatal unit from March 2019 to July 2022 and followed-up until hospital discharge were included. We excluded those whose delivery did not occur at the institution and/or NBs with a surgical diagnosis of complex gastroschisis (presence of stenosis, intestinal atresia, perforation, necrosis or volvulus according to the eviscerated content), born to mothers with a recent history of legal drug use (before hospitalization and delivery), HIV infection or human T-cell lymphotropic virus (HTLV).

Each mother of a newborn eligible for the study was approached and given the details about the research protocol, which included the randomization of the study groups, the milking procedure and the oropharyngeal colostrum administration. After parental informed consent was obtained, each newborn was randomly allocated to the intervention group or the control group. The NBs in the intervention group received 0.2mL of maternal colostrum administered directly into the oral mucosa during the first 48 hours of life for a period of 3 days with 3-hour intervals between each treatment. For operational reasons, the protocol of oropharyngeal colostrum administration at night was not applied.

The control group received the “simulated procedure”, which consisted of the researcher remaining with her hand inside the incubator, with an empty syringe, for a period similar to that of the intervention group.

Patients were randomized in blocks of four and stratified into two groups of gestational age: < 37 weeks and ≥ 37 weeks. Randomization by gestational age was possible because adequate prenatal care at the institution allows for the scheduling of elective surgeries, with follow-up of obstetric complications and prevention of damage to the intestinal loops. The randomization of patients to both study groups was performed through the website Randomization.com (http://randomization.com).

Blinding was applied to all health professionals of the neonatal unit involved in the study, as well as to the statistician responsible for data analysis.

Only the research member who performed the colostrum therapy and the lactary member knew to which group each newborn belonged.

All mothers were instructed on manual expression with aseptic techniques at the bedside. These procedures, information and guidelines on expressing milk are used for all mothers who do not breastfeed in the first hours of life, as recommended in the hospital routine.

The mothers received a sterile colostrum storage bottle, identified with the date, time and name of the newborn. The researcher remained by the mother’s side to assist her in this process. When manual milking was impossible, an electric pump (Pump Mini Electric-Medela®) was used, and its accessories were duly sterilized before each use.

Colostrum collection in the first 24 to 48 hours occurred in the ward where the mother was hospitalized (hosting room or pregnant women’s ward). The mothers were instructed and encouraged to massage and express the milk in the first 24 hours, every 2 or 3 hours, understanding that the process of stimulation of the breasts, during hospitalization and after hospital discharge, is essential for the success of milk production. Only two mothers were unable to express themselves by hand, requiring the use of an electric pump.

For the NBs randomized to the intervention group, the mother’s own colostrum was sent to the human milk handling site in a biological safety cabinet before being sent to the neonatal unit. For the NBs randomized to the control group (sham procedure), the colostrum was stored in refrigerators available in the hospitalization units and subsequently sent to the Milk Bank. There was no medical prescription for the intervention, and blinding was respected.

The protocol proposed by Rodriguez et al.^(11)^ was used. The technique for oropharyngeal colostrum administration consists of gently introducing a sterile 1mL syringe without a needle into the mouth of the NB along the right buccal mucosa (0.1mL) toward the oropharynx and repeating the procedure on the left buccal mucosa (0.1mL) without removing the syringe from the mouth of the NB.

The syringes were identified with the data of the NB (name, medical record, care unit and time of distribution), wrapped in autoclave adhesive tape (Missner® 19mm x 30m autoclave-proof adhesive tape) so that their contents could not be identified, sealed with a sterile universal colored syringe cap and personalized in a plastic bag for distribution.

A total of 0.2mL was administered every 3 hours during the day, totaling 0.8mL every 24 hours, the therapy was not conducted during the night due to operational reasons. The oropharyngeal administration of colostrum was only performed for 72 hours because, after delivery, the mothers remained in the hospital for approximately 2 to 3 days.

After the therapy was completed, the NB followed the routine of the neonatal unit, with a zero diet, until medical release. During the entire procedure, the NB remained monitored for oxygen saturation, heart rate and respiratory rate. An adverse event was the occurrence of any change in these parameters: heart rate > 200 or < 80 beats per minute, respiratory rate > 60 breaths per minute or oxygen saturation < 88%.

The following events were considered lost to follow-up: early neonatal death, use of treatment doses below 75% of the required amount (either due to nonavailability of maternal colostrum or clinical instability of the newborn) and maternal withdrawal from the study. Study suspension was considered if, after the interim analyses, the data revealed clear harms or benefits.

The beginning of enteral nutrition was determined by the physician responsible for the neonatal unit, and human milk was preferred, according to the protocol already followed in the institution. Breastfeeding was initiated when the NB was clinically stable, and the volume of orally administered diet by cup or bottle was approximately 25/30mL of human milk.

Data were collected from medical records, including demographic data and medical history during hospitalization until hospital discharge. The primary outcome measure was the number of days from birth to oral feeding (first sucking diet). The secondary clinical outcomes were the number of days without food, number of days on parenteral feeding, number of days until start of enteral tube feeding, number of days to reach full enteral feeding (100/120mL/kg/day), sepsis (positive blood culture) and total length of stay (until hospital discharge).

The sample size was calculated considering a significance level of 5% and power of 80% to show clinically relevant differences at the beginning of the oral diet. Based on the results of previous studies,^(11)^ the mean time to oral initiation in patients undergoing colostrum therapy was 69.86 ± 19.33 days, while in the placebo group, it was 55.83 ± 12.97 days. Patients were randomized to receive the intervention or “sham procedure” in a 1:1 ratio. Thus, the smallest sample size required was 22 patients in each treatment group.

Data were collected using the research form and the NB medical records. The Statistical Package for the Social Sciences (SPSS) software for Windows, version 13.0 (SPSS Incorporation, 1998), was used for data analysis with a significance level of 5%. Analysis of variance was performed for variables with a normal distribution, and nonparametric tests were performed for variables with a nonnormal distribution (Kruskal‒Wallis and Wilcoxon tests). Chi-square and Fisher’s exact tests were used for the analysis of categorical variables. For the comparison between groups, numerical or qualitative ordinal variables, the Wilcoxon-Mann‒Whitney U test was applied.

The Ethics Committee for Research on Human Beings approved this project under number 2,942,243 (CAAE: 99246718.4.0000.5269) on March 14, 2019. It was included in the Brazilian Clinical Trials Registry (REBEC - *Registro Brasileiro de Ensaios Clínicos*), U1111-1248-2063, and registered on March 27, 2020, http://www.ensaiosclinicos.gov.br/rg/RBR-3t3jvr/.

## RESULTS

**Figure 1:**
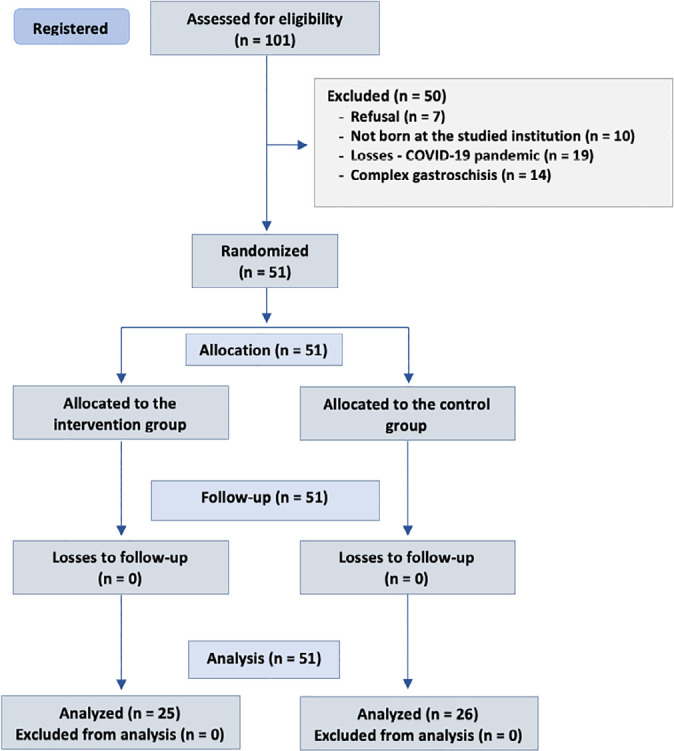
Study flowchart.

In the period from March 2019 to July 2022, 101 NBs were eligible, of which 50 were excluded, as shown in the study flowchart. Fifty-one newborns were randomized, 25 into the sham procedure group and 26 into the colostrum intervention group (Figure 1).

The maternal and neonatal demographic characteristics of the study population are described in table 1. We can observe that there was no statistically significant difference in any of the variables analyzed, and the groups were comparable in terms of entry variables.

**Table 1:** Maternal and newborn characteristics with simple gastroschisis in the neonatal period

	Control group (n = 26)				Intervention group (n = 25)				p value
	Mean ± SD	Median	Minimum	Maximum	Mean ± SD	Median	Minimum	Maximum	
Maternal age	19.6 ± 3.4	18	13	28	21.2 ± 4.1	22	13	30	0.11^*^
Number of prenatal consultations	5.5 ± 3.4	4.5	1	11	4.1 ± 2.8	4	1	10	0.51^*^
Time of mechanical ventilation (hour)	93.9 ± 92.8	48	8	288	86.6 ± 86.4	72	14	384	0.84^*^
Birth weight (g)	2,298 ± 479	2,319	1,420	3,110	2,429 ± 404	2,374	1,720	3,428	0.50^*^
Gestational age (weeks)	36.3 ± 1.6	37	32	38	36.4 ± 1.1	37	34	39	0.68^*^
	**n (%)**				**n (%)**				
Childbirth									
Vaginal	4 (15.38)				7 (28)				0.32†
Cesarean section	22 (84.62)				18 (72)				
Smoker	2 (7.7)				3 (12)				0.66†
1-minute Apgar score									
≥ 7	20 (80.7)				18 (76)				0.30†
< 7	6 (19.3)				7 (24)				
Sex									
Male	13 (53.8)				11(44%)				0.57†
Female	12(46.2)				14(56)				
Fetal distress	7 (27)				3 (12)				0.29†

* Kruskal-Wallis test; † Fisher's exact test. SD - standard deviation.

Regarding the surgical aspects of gastroschisis, there were also no significant differences between the groups. Most newborns did not use a silo (60%). When used, the average time of use was 4 days, which can be explained by the size of the defect, ranging from 1.5 to 5.0cm, or because of hemodynamic instability during the reduction of intestinal loops. Surgical correction occurred within the first 6 hours of life, with a median of 5 hours, but the size of the defect in centimeters was greater in the control group (Table 2).

**Table 2:** Surgical characteristics of infants with simple gastroschisis in the study population

	Control group (n = 26)				Intervention group (n = 25)				p value
	Mean ± SD	Median	Minimum	Maximum	Mean ± SD	Median	Minimum	Maximum	
Lifespan at first surgery (hour)	5.2 ± 4.2	5	1	18	5.0 ± 3.3	5	1	16	0.94^*^
Size of the defect L (cm)	2.7 ± 0.9	2.5	1.5	5.0	2.1 ±0.7	2.5	1.0	4.0	0.46^*^
Size of the defect T (cm)	2.7 ± 0.7	3.0	1.5	4.5	2.4 ±0.7	2.2	1.0	3.5	0.19^*^
Duration of silo use (days)	4.1 ± 2.0	4	1	7	3.6 ±1.4	3.5	2	6	0.59^*^
Previous gastric residue volume (mL)	12.8 ± 7.4	11	0	25.8	10.6 ±5.7	10	0	27.8	0.25^*^
Volume of gastric residue per day (mL)	3.2 ± 4.0	2.0	0	17.2	3.5 ± 3.1	3.0	0	10.0	0.40^*^
	**n (%)**				**n (%)**				
Use of silo	9 (34.6)				8 (32)				0.53†
Feeding discontinued after feeding initiation	8 (38.1)				13 (61.9)				0.15†

* Kruskal-Wallis test; †Fisher's exact test. SD - standard deviation.

According to the evaluation of the pediatric surgeon, the appearance of bowel loops was classified as “good” at birth in 73.1% of the NBs in the control group and 64% of the NBs in the intervention group (p = 0.55). Primary surgical closure was performed in 34 NBs, while 17 underwent staged closure, with the silo being the first surgical approach, with a median of 3 surgical approaches until completion of abdominal wall closure.

The onset of oral feeding in patients with simple gastroschisis in both groups occurred at a median of 15 days (Table 3), regardless of the volume of gastric residue on the day feeding was initiated, which ranged from no gastric residue to 17.2mL (Table 2).

**Table 3:** Neonatal outcome of infants with simple gastroschisis in the study population

	Control group (n = 26)				Intervention group (n = 25)				p value
	Mean ± SD	Median	Minimum	Maximum	Mean ± SD	Median	Minimum	Maximum	
Days on zero diet	19.5 ± 12.4	15.5	7	64	18.4± 11.7	16	4	54	0.73^*^
Days on total parenteral nutrition	25 ± 13.6	22	9	69	28.9 ± 26.5	21	7	121	0.74^*^
Days before introduction to enteral feeding†	4.73 ± 6.0	0	0	16	8.4 ± 15.1	0	4	68	0.81^*^
Days before oral diet (sucking)	19.8 ± 7.0	15	7	66	17.1 ± 10.1	15	4	52	0.75^*^
Days before complete oral feeding (without tube)‡	23.0 ± 19.7	23.5	0	76	26.8 ± 31.1	20	0	125	0.87^*^
Days before hospital discharge	38.2 ± 16.6	36	15	81	44.8 ± 42.4	33	16	213	0.54^*^
Sepsis, n (%)	11 (42.3)				9 (36)				0.77^*^

Complete enteral diet - reaches a dietary value of 100 mL/kg/day.

* Kruskal-Wallis test; † enteral feeding = control group (n = 11), intervention group (n = 9); ‡Suction only = control group (n = 19), intervention group (n = 18). SD - standard deviation.

Regarding the reason for feeding interruption, as shown in table 2, the most frequent cause in the control group was abdominal distension (n = 5; 62.5%), followed by vomiting (n = 3; 37.5%). In the intervention group, 11 newborns presented with the following main reasons: vomiting (n = 5; 45.4%); abdominal distention (n = 4; 36.4%); gastric residue (n = 1; 9.1%) and clinical alteration (n = 1; 9.1%). No adverse effects were observed.

Table 3 presents the main clinical outcomes of the study population, showing that the groups did not differ from each other. However, although there was no statistically significant difference in these results, oropharyngeal colostrum administration did not provide protection for the intervention group, nor did it reduce the risk of sepsis and benefits to facilitate enteral feeding.

## DISCUSSION

Oropharyngeal administration of breast milk has been proposed as a viable alternative to stimulate the immature neonatal immune system and provide protection against infections.^(4,11)^

Limited scientific evidence suggests that oropharyngeal colostrum administration, started within the first 48 hours of life, does not reduce the risk of necrotizing enterocolitis, late-onset infection or death in very small and very low birth weight infants.^(4,17)^

However, there is an investigation in the study by Ouyang et al.^(2)^ that showed a beneficial effect in the administration of 0.4 mL of oral colostrum in NBs with gestational age ≤ 32 weeks. According to that study, colostrum therapy may have a potential effect in reducing the incidence of necrotizing enterocolitis, late-onset sepsis and severe intraventricular hemorrhage and shortening the time to achieve full enteral nutrition.

The lack of robust evidence to support this practice is limited by the lack of studies with adequate power to evaluate the effects of this intervention on clinically relevant outcomes, especially reducing the incidence of early-onset or late-onset infection and necrotizing enterocolitis, which compromises neonatal outcomes.^(13-15,17)^

This study is a randomized, double-blind, controlled clinical trial by a “sham procedure”, which showed that there were no significant differences between the two groups regarding oropharyngeal colostrum administration in relation to baseline characteristics and clinical outcomes.

Other published studies show differences regarding dosages, frequency of administration, duration of intervention, type of milk, benefit population (weight and gestational age); some studies even included oropharyngeal colostrum as part of a feeding protocol, hindering the standardization and applicability of findings.^(11,14)^

Studies with preterm infants observed good clinical results, favorable to the colostrum group, regarding the decrease in the time required to obtain complete enteral feeding, but there was no reduction in the length of hospital stay. This result is similar to that found in the study by Seigel et al.,^(18)^ with a longer hospital stay among newborns who received colostrum therapy.^(4,12)^

The studies that propose the oropharyngeal administration of colostrum in the neonatal hospital context involve NBs with preterm births and are not yet being performed in NBs with syndromes or malformations such as gastroschisis.^(8-10)^

Based on this premise and the scarcity of studies on the use of colostrum therapy in NB patients with gastroschisis, this protocol was elaborated, constituting an important stage in the accomplishment of the research, allowing a new result and decision-making regarding the therapy. After the development and execution of this document, no difficulties were observed in conducting the study, as suggested in other studies.^(11,13,14)^

In our study, the colostrum therapy protocol for gastroschisis was initiated between 48 and 72 hours after birth, with good tolerance by the population, and no adverse events were observed, except in one NB who showed a drop in oxygen saturation (88%), with spontaneous return, and exclusion from the study was not necessary.

The oropharyngeal administration of colostrum was only conducted during the first 3 days of life of the newborn, and even then, all the newborns were followed up until hospital discharge.

Regarding dosages, frequency of administration, duration of intervention, type of milk, and benefit population (weight and gestational age), the studies are divergent, making standardization and applicability in neonatal units difficult.

Most studies reported the value of 0.2mL as the administered dosage, which was also adopted for this study, with a frequency of every 3 hours for 3 consecutive days. In the literature, the studies that adopted the same volume and frequency as our study were the studies by Lee et al.^(19)^ and Glass et al.;^(20)^ however, the latter involved colostrum therapy using a *swab*, not a syringe, with variations in administration times and results.

One of the limitations of the present study is that the frequency, the dosage and the period of performance of the protocol of oropharyngeal colostrum administration were based on studies with preterm NB who had no malformations. It might be interesting to conduct studies with larger colostrum volumes and higher frequency of colostrum administration.

The study population also differed from other studies regarding birth weight. As of 2018, the Ministry of Health, with the document on the Kangaroo Handbook, recommended that the dosage administered should be determined by weight range, with dose values ranging from 0.2mL to 0.6mL, and associating colostrum therapy with the Kangaroo Method, a standardized protocol for better neonatal outcomes.

This guideline may be used in future studies with a larger number of participants.

Our initial hypothesis was that NBs with simple gastroschisis could benefit from oropharyngeal colostrum administration with regard to early initiation of oral feeding.

The applicability of this strategy is related to the importance of the oral mucosa and oropharynx for immune development and, consequently, of the gastrointestinal tract.^(21^.^22)^ This may also be beneficial for this population, even though the results of the present study did not show differences between the groups with respect to the primary outcomes.

Dysmotility is a type of intestinal dysfunction expected in gastroschisis. However, the results of our study showed that the introduction of enteral feeding with human milk occurred with a median of 15 days, corroborating the findings of other studies.^(23-25)^

In our study, intestinal dysmotility, found in 8 NBs in the control group and in 13 in the intervention group, was not observed during the use of colostrum therapy. It was related to episodes of vomiting, abdominal distension and difficulty in diet progression, which made it necessary to suspend the diet or reintroduce it slowly with human milk or hydrolyzed formula. Newborns who presented feeding reactions after initiating enteral feeding were not excluded from the analysis.

Oropharyngeal colostrum is not included in the nutritional protocol for simple gastroschisis at this institution. The practice is to feed newborns breast milk in the first days of life. Findings reinforce the prior knowledge of the importance of early nutrition, especially with human milk during the entire hospitalization period.

This was one of the first studies to apply the protocol for oropharyngeal colostrum administration to neonates with malformations, and our study had some considerable limitations.

The first difficulty encountered in conducting the present study was regarding the input parameters for calculating the sample size because there are no publications on the use of colostrum therapy in NBs with gastroschisis, so we used studies of clinical trials with preterm NB without malformations as a basis. Studies that used breast milk in NBs with gastroschisis did not use colostrum, except for those that suggested a protocol of oral hygiene with colostrum or breast milk.^(16,26)^

This study was conducted at a reference center for comprehensive care, with preand postnatal follow-up and clinical neonatal and surgical management for NBs. Thus, the bias related to births in another health unit were reduced. However, this single study center may also be a limitation of the study, even considering the increase in the prevalence of gastroschisis in recent years.

At the study institution, the pregnant women are welcomed and scheduled for several prenatal consultations with specialists in medicine, nutrition, genetics, social work, milk bank, psychology. In some of these consultations, the women were approached to participate in this study. Concomitantly, the study was explained in collegiate groups, in meetings with supervisors and managers (neonatal and neonatal surgery) and in the sectors where the mother was after delivery (rooming-in and ward for pregnant women) and during shift changes because the project was not a hospital routine.

Among the hospital units, there is surgical mapping for monitoring births; however, even with the knowledge of this schedule, there was still a loss of NBs in the study because strict controls were not followed.

During the coronavirus disease 2019 (COVID-19) pandemic, more specifically the period from April to August 2020, the neonatal unit underwent a rigorous cohort process to control the flow of employees, and research in this sector was suspended. These circumstances led to the interruption of the project during this period and the noninclusion of 15 eligible NBs for the study.

## CONCLUSION

This study did not observe the efficacy of oropharyngeal administration of maternal colostrum in gastroschisis regarding clinical outcomes between the two groups.

Despite this result, the intervention was feasible for this population and its administration was safe; it be a treatment for all newborns with gastroschisis.

The greatest contribution of this study was maternal involvement with regard to the preparation of mothers for lactation stimulation by expressing at the bedside, since removing colostrum is an early incentive and stimulus for breastfeeding.
